# Identification of potential core genes in idiopathic pulmonary arterial hypertension: An observational study highlighting the role of VEGFA

**DOI:** 10.1097/MD.0000000000045398

**Published:** 2025-10-24

**Authors:** Yingchuan Zhou, Lifang Xu, Ailing Ou, Shuilian Gan, Wenze Deng

**Affiliations:** a Department of Ultrasound, The Second Affiliated Hospital of Guangxi Medical University, Nanning, China.

**Keywords:** bioinformatics analysis, differentially expressed genes, GEO data, hub genes, idiopathic pulmonary arterial hypertension, *VEGFA*

## Abstract

Idiopathic pulmonary arterial hypertension (IPAH) is a progressive disease characterized by unexplained pulmonary vascular resistance, which can lead to persistent pulmonary arterial hypertension and eventually right heart failure. A targeted therapy for IPAH that can effectively reduce pulmonary artery pressure and improve survival and prognosis is urgently required. This study aimed to identify potential core genes and pathways involved in the pathogenesis of IPAH through a bioinformatics approach. Two publicly available gene expression datasets (GSE15197 and GSE130391) from the Gene Expression Omnibus were analyzed in this observational study, encompassing 22 IPAH and 17 control lung specimens. The GEO2R tool was employed to identify differentially expressed genes. Gene Ontology and Kyoto Encyclopedia of Genes and Genomes pathway databases were employed for the functional enrichment analysis of the identified differentially expressed genes. STRING and Cytoscape were used to construct and visualize a protein–protein interaction network, respectively, for the identification of hub genes. A total of 159 genes were identified, of which 56 were downregulated and 103 were upregulated. Their biological functions mainly focus on negative regulation of transcription (DNA-templated), positive regulation of RNA polymerase II promoter transcription, zinc ion binding, and protein heterodimerization activity. Enrichment mapping revealed that the phosphatidylinositol 3-kinase–protein kinase B axis and cancer pathways constituted the central regulatory networks for the differentially expressed gene cohort. Ten hub genes were identified, including the notably downregulated core gene vascular endothelial growth factor A. While this STROBE-compliant study was computational in nature, the 10 hub genes identified present promising candidates for future exploration in IPAH prevention, diagnostic strategies, and therapeutic development. These findings should be interpreted as generating hypotheses that necessitate confirmation through rigorous experimental validation to establish their biological significance. Notably, vascular endothelial growth factor A emerged as a particularly intriguing and unanticipated differentially expressed gene, with its downregulation potentially representing a distinctive feature of IPAH among pulmonary hypertension subtypes.

## 1. Introduction

Idiopathic pulmonary arterial hypertension (IPAH) is a progressive, fatal disorder characterized by elevated mean pulmonary arterial pressure, ultimately causing premature mortality via right ventricular failure.^[1]^ In IPAH, the increased vascular resistance is secondary to vascular remodeling, cellular proliferation, inflammation, vasoconstriction, and in situ thrombosis.^[2]^ Ambrisentan, bosentan, and sildenafil are standard therapies for pulmonary arterial hypertension (PAH), with sildenafil demonstrating superior survival benefits and enhanced suitability for long-term management compared with endothelin receptor antagonists.^[3]^ This may be because the nitric oxide signaling pathway demonstrates superior therapeutic efficacy and safety to endothelin-targeted approaches for managing pulmonary hypertension.^[3]^ Despite therapeutic advancements in pulmonary hypertension, current therapies fail to completely eliminate IPAH, with persistently short post-diagnosis survival.^[4]^ Although some progress has been made in current drug research for pulmonary hypertension, studies to explore potential therapeutic targets and effective treatments are needed to reduce the mortality of IPAH.

The function of angiogenesis and vascular endothelial growth factor (VEGF) signaling in PAH presents a well-documented yet unresolved paradox. Despite its fundamental role in angiogenesis, the VEGF/vascular endothelial growth factor A (VEGFA) axis has also been closely linked to the disruptive vascular remodeling that constitutes a defining pathological feature of PAH.^[5]^ VEGF signaling maintains crucial dialogs with other cardinal pathways in PAH, including nitric oxide and endothelin systems, thereby modulating key endothelial functions such as cellular survival, proliferation, and membrane permeability.^[6]^ The specific expression profile and pathological role of VEGFA in IPAH specifically, as distinct from other PAH forms, are not yet fully defined, with sometimes disparate findings across studies highlighting the need to more precisely delineate its cell-type-specific functions.^[7]^ Given this mechanistic rationale and persistent controversy in the literature, VEGFA was selected as a high-priority target for detailed scrutiny in our investigation.

Gene expression microarrays have been widely used to collect and analyze gene expression profiling data for various cancers, because they are efficient and cost-effective. Bioinformatics analysis is also a potent tool for detecting possible pathomechanisms of diseases from the perspective of gene regulation. Previous studies aimed at identifying differentially expressed genes (DEGs) in IPAH have provided inconsistent results, most likely because of the use of different source tissues. Therefore, only data from lung tissue samples were analyzed in the present study.

Two original microarray datasets with information on IPAH (GSE15197 and GSE130391) are available from the Gene Expression Omnibus (GEO) database of the National Center for Biotechnology Information (https://www.ncbi.nlm.nih.gov). These 2 microarray datasets include data for a total of 39 specimens, 22 specimens of lung tissue from IPAH patients, and 17 specimens of normal lung tissue. Two microarray datasets were systematically analyzed using comprehensive bioinformatics approaches to identify DEGs. In the present study, we downloaded these 2 microarray datasets and identified DEGs via bioinformatics analysis. The biological functions and interactions of DEGs were further investigated. Conceived as a hypothesis-generating in silico exploration, this study provides crucial insights and prioritizes key candidate genes and pathways in IPAH. It is imperative to emphasize that these findings are fundamentally preliminary and necessitate rigorous experimental validation to definitively establish their biological mechanisms and therapeutic relevance.

## 2. Methods

### 2.1. Ethical statement

This retrospective analysis of publicly available, de-identified genomic data from the GEO repository did not require institutional review board approval or informed consent, as confirmed by the Institutional Review Board of The Second Affiliated Hospital of Guangxi Medical University.

### 2.2. Acquisition of gene expression profile data

Gene expression profiles (accession numbers GSE15197 and GSE130391) associated with PAH were systematically retrieved from the National Center for Biotechnology Information GEO database for a comprehensive analysis. The GSE15197 dataset, derived from the Agilent-014850 platform (GPL6480), comprises 18 IPAH and 13 control lung tissue specimens for comparative genomic profiling. The GSE130391 dataset, generated via the Affymetrix HG-U133 Plus 2.0 Array (GPL570), included matched lung tissue specimens from 4 IPAH patients and 4 healthy controls for transcriptional comparison. The tissue source for both chips was lung tissue, and the specific information is presented in Table 1.

**Table 1:** Core characteristics of 2 microarray datasets systematically acquired from the Gene Expression Omnibus (GEO) repository.

Reference	Sample source	GEO	Platform	No. of IPAH samples	No. of control samples
Rajkumar et al (2010)	Lung tissue	GSE15197	GPL6480	18	13
Hemnes et al (2019)	Lung tissue	GSE130391	GPL570	4	4

IPAH = idiopathic pulmonary arterial hypertension.

### 2.3. Recognition of DEGs in IPAH

Differential expression analysis between IPAH and normal lung tissues was performed independently for each dataset (GSE15197: Agilent-014850, GPL6480; GSE130391: Affymetrix HG-U133 Plus 2.0, GPL570) using the GEO2R online tool (https://www.ncbi.nlm.nih.gov/geo/geo2r/). To circumvent the substantial batch effects between the disparate Agilent and Affymetrix platforms, we analyzed each dataset independently instead of applying correction methods. GEO2R, which utilizes the limma R package, applied quantile normalization to processed data. The false discovery rate was controlled using the Benjamini–Hochberg method. Significantly differentially expressed genes were identified using thresholds of adjusted *P*-value < .05, |log_2_FC| ≥ 1. Common genomic loci across both datasets were determined through the systematic application of the Venn diagram analytical module (Venn Diagram Webtools platform: bioinformatics.psb.ugent.be/webtools/Venn/) for multi-dataset comparative visualization.

### 2.4. Gene Ontology enrichment analysis and Kyoto Encyclopedia of Genes and Genomes pathway analysis

Functional enrichment analysis was conducted to interpret the DEGs in the context of Gene Ontology (GO) and Kyoto Encyclopedia of Genes and Genomes (KEGG) pathways. GO offers a structured categorization of gene functions into biological processes, molecular functions, and cellular components, while KEGG facilitates the examination of genes within known biological pathways. These analyses were carried out using DAVID bioinformatics resources (version 6.8). Significantly enriched terms were identified based on a false discovery rate-corrected *P*-value (Benjamini–Hochberg) below .05, along with a minimum of 10 genes per term. Result visualization was achieved with the GOplot package in R, generating integrated plots from both GO and KEGG annotation datasets.

### 2.5. Topological mapping of protein–protein interaction architectures coupled with precision characterization of pivotal network modules

Protein–protein interaction (PPI) network analysis was performed for the identified DEGs using the String software (http://string-db.org/). String software (version 11.0) was used to construct and visualize the PPI network with a minimum required interaction score of >0.400 and disconnected nodes within the hidden network. We pruned all isolated proteins (those lacking any interactions) from the network to confine our subsequent analysis solely to interconnected complexes. The resulting network was subsequently analyzed in Cytoscape, where hub gene identification and ranking were performed employing the Edge Percolated Component algorithm via the cytoHubba plugin (www.cytoscape.org/). Based on EPC ranking, the 10 highest-scoring nodes were defined as pivotal hub genes and advanced to subsequent analytical stages.

## 3. Results

### 3.1. Data characteristics

Based on 2 publicly available microarray datasets, GSE15197 and GSE130391, this work profiled the transcriptomes of IPAH and control lung tissues. The cohort sizes were 31 (18 IPAH/13 control) and 8 (4 IPAH/4 control) for the 2 datasets, the specifics of which are comprehensively summarized in Table 1.

### 3.2. Screening of DEGs

**Figure 1.**
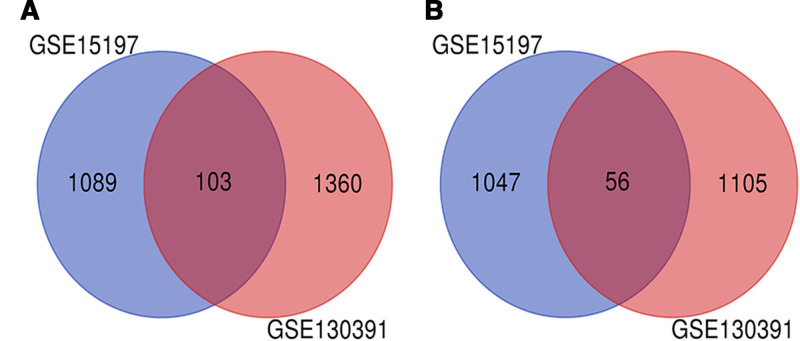
Venn diagram. DEGs were screened according to the criteria of an adjusted *P*-value < .05 and |log_2_FC| ≥ 1 among the gene expression profiles GSE15197 and GSE130391. The GSE15197 dataset included 18 specimens of lung tissue from IPAH patients and 13 specimens of normal lung tissue. The GSE130391 dataset included 4 specimens of lung tissue from IPAH patients and 4 specimens of normal lung tissue. (A) Upregulated genes, and 103 genes overlapped between the upregulated genes of these 2 datasets based on the criteria of an adjusted *P*-value < .05 and |log_2_FC| ≥ 1. (B) Downregulated genes, and 56 genes overlapped between the downregulated genes of these 2 datasets based on the criteria of an adjusted *P*-value < .05 and log_2_FC ≦ –1. DEG = differentially expressed gene, IPAH = idiopathic pulmonary arterial hypertension.

Applying thresholds of an adjusted *P*-value < .05 and |log_2_FC| ≥ 1, the GSE15197 dataset analysis revealed 2295 DEGs, comprising 1192 significantly upregulated and 1103 downregulated transcripts. Employing the same criteria, analysis of the GSE130391 dataset detected 2624 DEGs, with 1643 genes exhibiting significant upregulation and 1161 displaying downregulation. A web-based Venn diagram was employed to identify the intersection of DEGs across datasets, yielding a core set of 159 consistently dysregulated transcripts (Fig. 1).

Summary of DEGs:

GSE15197: 2295 DEGs (1192 up, 1103 down).

GSE130391: 2624 DEGs (1643 up, 1161 down).

Intersection: 159 common DEGs (103 up, 56 down).

Table 2 presents the log_2_ fold changes along with their 95% confidence intervals for the major hub genes, as determined from the higher-power GSE15197 cohort.

**Table 2:** The log_2_ fold changes and their 95% confidence intervals for the top hub genes identified from the GSE15197 dataset.

Gene symbol	log_2_FC	95% CI lower	95% CI upper	FDR-adjusted *P*-value
VEGFA	−1.11	−1.51	−0.71	.000245
HUWE1	2.39	1.19	3.59	.00376
MDM2	1.09	0.43	1.75	.0137
PTGS2	−1.69	−2.63	−0.75	.00789
KMT2A	1.08	0.53	1.63	.0046
MCL1	−1.08	−1.66	−0.50	.00609
KMT2C	1.22	0.63	1.81	.00233
RNF6	−1.28	−1.78	−0.78	.000521
KAT6A	1.55	0.64	2.46	.0113
HERC2	1.8	0.97	2.63	.00188

The 95% confidence intervals (CIs) were calculated based on the standard error derived from the *t*-statistic (SE = log_2_FC / t ). A CI that does not cross zero (e.g., both limits are positive or both are negative) indicates that the change in gene expression is statistically significant at the 5% level, which is consistent with the adjusted *P*-value < .05.

FC = fold change, FDR = false discovery rate.

This core set of 159 intersecting differentially expressed genes (103 upregulated, 56 downregulated) reflects consistent transcriptional alterations in IPAH lungs and underpins all subsequent functional and topological explorations. The marked bias toward upregulated transcripts indicates a broadly activated transcriptional landscape, potentially driving disease progression.

### 3.3. Functional enrichment analysis of DEGs

#### 3.3.1. GO enrichment analysis of DEGs

**Figure 2.**
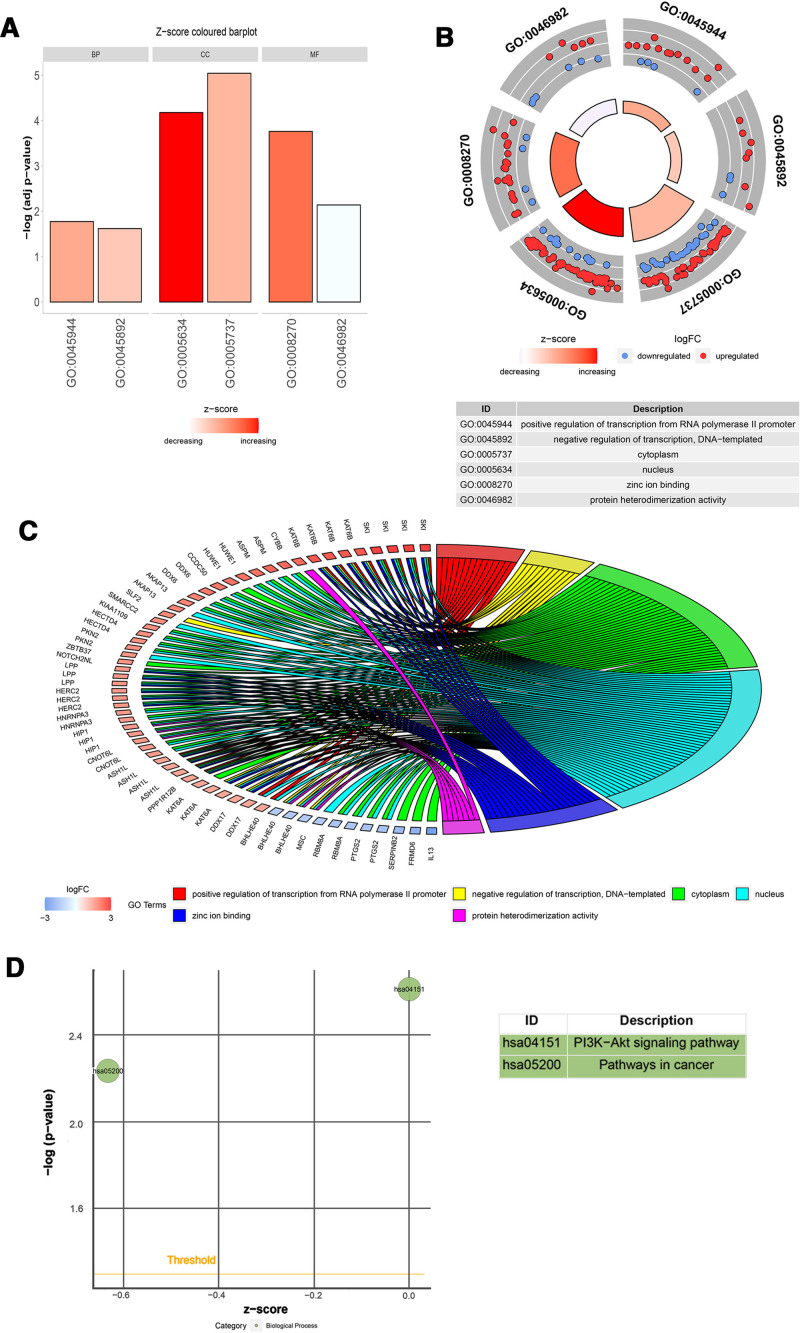
Distribution of DEGs in IPAH according to different enriched GO terms and enriched KEGG pathways. (A) Two of the most significantly enriched GO terms for DEGs in each diverse functional groups. (B) Circular visualization of DEGs enrichment analysis results. The scatter plot in outer circle displays the log_2_FC of the assigned genes in each term. Red circles indicate up, and blue circles indicate down. (C) Distribution of DEGs in diverse enriched GO functions. DEGs were screened based on the criterion of a |log_2_FC| ≥1.5. In panels A–C, the same enriched GO terms were selected in each diverse functional group based on the criteria of a *P*-value < .05 and gene count ≥ 10. (D) Bubble plot of the 2 most significantly enriched KEGG pathways. A *P*-value < .05 and gene count ≥ 10 were applied as the criteria. DEG = differentially expressed gene, GO = Gene Ontology, IPAH = idiopathic pulmonary arterial hypertension, KEGG = Kyoto Encyclopedia of Genes and Genomes.

GO enrichment analysis of DEGs in IPAH was performed using DAVID. Functional annotation analysis demonstrated a significant enrichment of DEGs in transcriptional regulation pathways, specifically: bidirectional modulation of RNA polymerase II-mediated transcription, DNA-dependent transcriptional activation/repression, and signal transduction mechanisms. In the molecular function component, DEGs were enriched in the Golgi apparatus, zinc ion binding, protein binding, protein heterodimerization activity, poly(A) RNA binding, protein homodimerization activity, and nucleic acid binding. In the cell composition component, DEGs were mainly enriched in the cytoplasm, nucleus, nucleoplasm, protein complexes, membrane, and Golgi apparatus. Table 3 summarizes the full set of statistically enriched GO terms. Some of the significantly enriched GO terms for DEGs in IPAH are shown in Figure 2A–C. These results indicated that the most significantly upregulated genes were enriched in the nucleus, cytoplasm, positive regulation of transcription from the RNA polymerase II promoter, and zinc ion binding. In contrast, the most significantly downregulated genes were enriched in the cytoplasm, nucleus, and protein heterodimerization activity.

**Table 3:** Enriched Gene Ontology terms prioritized from the IPAH DEG signature.

Category	GO ID	Count	FDR-adjusted *P*-value
BP	GO:0045944	16	.016837
BP	GO:0045892	10	.024016
BP	GO:0045893	10	.028641
BP	GO:0007165	17	.032456
BP	GO:0000122	12	.038501
CC	GO:0005737	68	9.01E−06
CC	GO:0005634	67	6.62E−05
CC	GO:0005654	38	.001183
CC	GO:0043234	10	.006294
CC	GO:0016020	28	.016823
CC	GO:0005794	13	.046644
MF	GO:0008270	24	1.73E−04
MF	GO:0005515	93	.0047
MF	GO:0046982	11	.007241
MF	GO:0044822	19	.008717
MF	GO:0042803	14	.010789
MF	GO:0003676	16	.023489

GO ID descriptions: Biological Process (BP): GO:0045944 (positive regulation of transcription from RNA polymerase II promoter), GO:0045892 (negative regulation of transcription, DNA-templated), GO:0045893 (positive regulation of transcription, DNA-templated), GO:0007165 (signal transduction), GO:0000122 (negative regulation of transcription from RNA polymerase II promoter). Cellular Component (CC): GO:0005737 (cytoplasm), GO:0005634 (nucleus), GO:0005654 (nucleoplasm), GO:0043234 (protein complex), GO:0016020 (membrane), GO:0005794 (Golgi apparatus). Molecular Function (MF): GO:0008270 (zinc ion binding), GO:0005515 (protein binding), GO:0046982 (protein heterodimerization activity), GO:0044822 (poly(A) RNA binding), GO:0042803 (protein homodimerization activity), and GO:0003676 (nucleic acid binding). Significantly enriched terms were identified using a threshold of FDR adjusted *P*-value < .05.

FDR = false discovery rate, GO = Gene Ontology.

#### 3.3.2. KEGG pathway analysis of DEGs

KEGG pathway enrichment analysis revealed that the phosphatidylinositol 3-kinase–protein kinase B signaling pathway and pathways in cancer were the most prominently enriched pathways among DEGs detected in the dual microarray datasets, as summarized in Table 4 and illustrated in Figure 2D.

**Table 4:** Statistically significant overrepresented KEGG signaling pathways of DEGs.

Term	Description	FDR-adjusted *P*-value
hsa04151	PI3K-Akt signaling pathway	.003
hsa05200	Pathways in cancer	.006

Significantly enriched pathways were identified using a false discovery rate (FDR)-adjusted *P*-value threshold of <.05. Analysis was performed on the 159 common differentially expressed genes (DEGs) using the DAVID bioinformatics platform.

KEGG = Kyoto Encyclopedia of Genes and Genomes, PI3K-Akt = phosphatidylinositol 3-kinase–protein kinase B.

### 3.4. PPI network construction and hub gene identification

**Figure 3.**
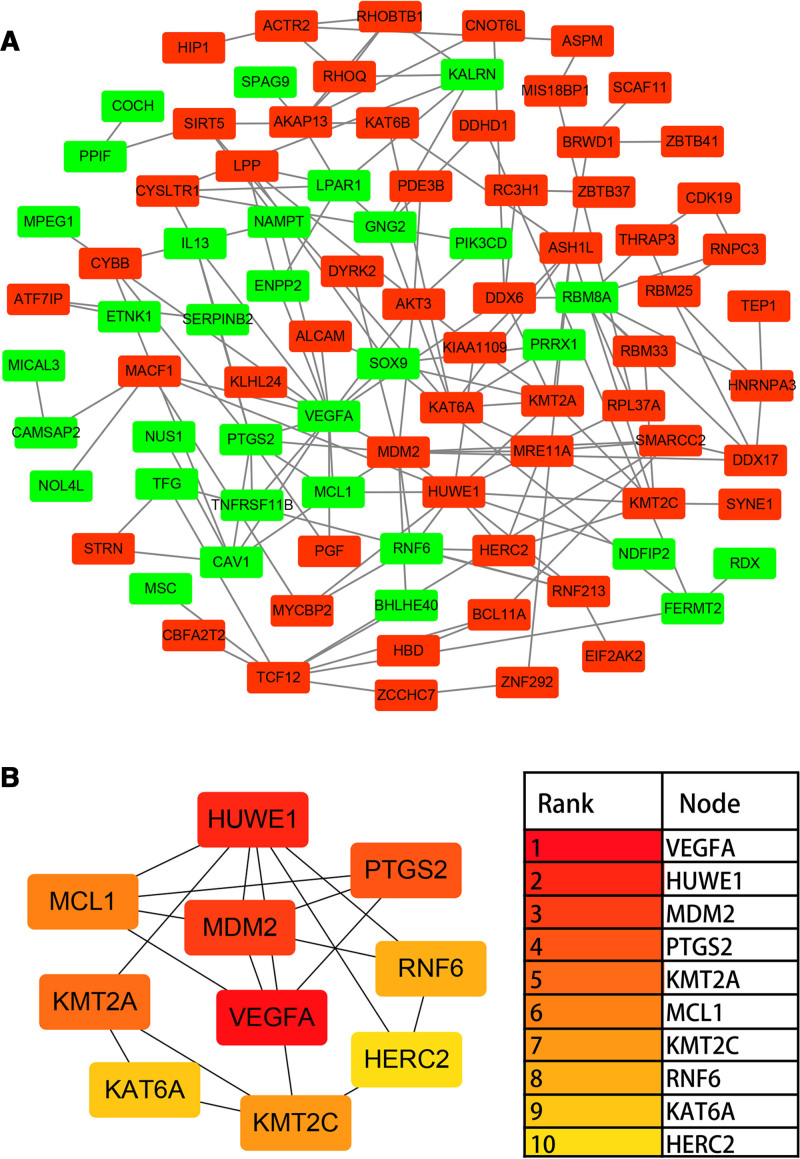
Protein–protein interaction network and top 10 hub genes derived from differentially expressed genes in IPAH. (A) The network was generated from the STRING database with a medium confidence threshold (0.400) and further analyzed in Cytoscape. Nodes in red and green denote up- or downregulated genes, respectively. (B) Hub genes were identified as the most influential nodes according to the Edge Percolated Component (EPC) metric using the CytoHubba plugin. The red color intensity of each node corresponds to its ranking, with darker shades indicating greater importance. IPAH = idiopathic pulmonary arterial hypertension.

Protein interactions among the DEGs were predicted with the Search Tool for the Retrieval of Interacting Genes (STRING) database (https://string-db.org/) and analyzed using Cytoscape software and the cytoHubba plugin. The minimum filtering ratio for PPI pair extraction was 0.4. The top 10 hub genes were identified and ranked using the Edge Percolated Component method (Fig. 3): vascular endothelial growth factor A (*VEGFA*), HECT, UBA, and WWE domain-containing E3 ubiquitin protein ligase 1 (*HUWE1*), mouse double minute 2 homolog (*MDM2*), prostaglandin-endoperoxide synthase 2 (*PTGS2*), lysine methyltransferase 2A (*KMT2A*), myeloid cell leukemia 1 (*MCL1*), lysine methyltransferase 2C (*KMT2C*), ring finger protein 6 (*RNF6*), lysine acetyltransferase 6A (*KAT6A*), and HECT and RLD domain-containing E3 ubiquitin protein ligase 2 (*HERC2*). Four hub genes were downregulated in IPAH (*VEGFA, PTGS2, MCL1*, and *RNF6*), while 6 hub genes were upregulated (*HUWE1, MDM2, KMT2A, KMT2C, KAT6A*, and *HERC2*).

## 4. Discussion

Leveraging publicly available GEO data, this in silico study identified 159 DEGs and 10 high-priority hub genes, including *VEGFA*, as potential key drivers in IPAH pathogenesis. While this computational approach provides a valuable, hypothesis-generating framework, its scope is circumscribed by the original cohort sizes and technical heterogeneity of the source datasets. Consequently, these predictive findings remain preliminary and mandate rigorous experimental validation in appropriate model systems and expanded clinical cohorts to ascertain their biological and translational relevance.

The *VEGFA* gene, which encoded *VEGFA* protein, has closely linked to PAH in previous studies and even shown to be a promising indicator for the diagnosis of PAH.^[5]^ *VEGFA* was also found to be upregulated in pulmonary hypertension and suggested as a potential biomarker, based on studies in in-vitro cell culture models, peripheral blood samples, and lung tissue samples.^[8–10]^ In contrast, the present study revealed that *VEGFA* was significantly downregulated in the lung tissues of patients with IPAH. The decreased *VEGFA* expression may have aggravated pulmonary vascular remodeling and increased the degree of tissue hypoxia, which is consistent with our findings.^[5]^ In addition, Campbell et al^[11]^ demonstrated that cell-based *VEGFA* gene transfer is a practical method for preventing and delaying the progression of pulmonary hypertension in a monocrotaline model; thus, targeting VEGFA demonstrates therapeutic potential for pulmonary hypertension intervention. One explanation for the discrepancy in the up- or downregulation of *VEGFA* in IPAH may be that IPAH has unique gene expression characteristics and pathophysiological mechanisms that could distinguish it from other subtypes of pulmonary hypertension.^[12,13]^ Because *VEGFA* is a key promoter of angiogenesis, overexpression of *VEGFA* is associated with poor outcomes in many cancers, including lung and prostate cancers.^[14–16]^ Vascular remodeling resulting in a reduction in the quality and number of pulmonary vascular channels is the fundamental pathogenesis of increased vascular resistance.^[2]^ Considering all this evidence together, we found that vascular remodeling and reduced *VEGFA* expression in the lung tissue of patients with IPAH are not contradictory, rather the lack of new blood vessel formation may be a potential cause of the enhanced degree of hypoxia and accelerated vascular remodeling.^[11]^ Findings from this study highlight the dysregulation of *VEGFA* signaling in IPAH, indicating that its function should be prioritized in future research. Subsequent work is needed to evaluate its candidacy as a biomarker and assess the therapeutic promise of targeting this pathway.

The elevated expression of MDM2 in IPAH lungs merits attention in light of its known functions in driving vascular cell proliferation and neointimal hyperplasia.^[17,18]^ This may underlie the hyperproliferative vascular remodeling that defines IPAH. Shridhar et al^[19]^ and Su et al^[20]^ revealed that *MDM2* antagonists suppress hyperplastic activity and cellular motility in vascular smooth muscle cells, decrease neointimal hyperplasia and vascular remodeling, and can be targeted for the treatment of hyperproliferative diseases. Consistently, we observed *MDM2* overexpression in patients with IPAH. *MDM2* also promotes tissue inflammation by regulating mitochondrial damage, and *MDM2* inhibitors have effective anti-inflammatory effects both in vitro and in vivo.^[21,22]^ These findings nominate *MDM2* as a novel factor in IPAH, prompting further investigation to define its mechanistic and therapeutic roles.

While the upregulation of HERC2 is a novel finding in IPAH with no established link to the disease, its known interaction with MDM2^[23,24]^ arises the possibility that it could function as a nexus within a broader regulatory network influencing cell fate decisions in the pulmonary vasculature. The complex composed of *MDM2*, oligomeric p53, *HERC2*, and NEURL4 regulates the transcriptional activity of p53, with *HERC2* playing a key role in the regulation of the p53–*MDM2* loop.^[23,24]^ Upon knockdown of *HERC2*, the mRNA expression of *MDM2* is reduced via inhibited activation of the *MDM2* promoter, eventually leading to a decrease in the *MDM2* protein level.^[24]^ Although overexpression of endothelial Sirtuin-1 in the carotid arteries of mice deficient in endothelial nitric oxide synthase prevented adverse arterial remodeling and alleviated hypertension, downregulation of *HERC2* expression eliminated this beneficial effect.^[25]^ The mechanism of *HERC2* activity in IPAH requires further investigation based on the controversial results of current studies regarding *HERC2* and arterial remodeling.

While *PTGS2* is often overexpressed in cancer and acts in a pro-inflammatory capacity,^[26]^ its expression was significantly reduced in IPAH. This finding is consistent with reports that *PTGS2* deficiency exacerbates disease in rodent models of hypoxic pulmonary hypertension,^[27,28]^ implying a protective role for this enzyme in the pulmonary vasculature. Xing et al^[29]^ demonstrated that BUR1, a novel piperidine, can significantly reduce pulmonary artery pressure and right ventricular systolic pressure, thereby preventing right ventricular hypertrophy and peripheral pulmonary artery thickening primarily by inducing the expression of bone morphogenetic protein 2 (*BMP2*) and *PTGS2* in the SU/Hy rat model. Consistently, *PTGS2* knockout mice develop severe pulmonary hypertension under chronic hypoxia via enhanced pulmonary vascular remodeling characterized by proliferation and exaggerated contraction of collagen matrices by pulmonary artery smooth muscle cells, suggesting that *PTGS2* deficiency is harmful in mice with hypoxia-induced pulmonary hypertension.^[27,29]^ Beyond pulmonary vascular remodeling, *PTGS2* deficiency or drug inhibition has been correlated with enhanced platelet aggregation and intravascular thrombus formation under chronic hypoxic conditions in rodent models of hypoxia-triggered pulmonary hypertension.^[28,30,31]^ This investigation demonstrated marked *PTGS2* reduction in pulmonary tissues from IPAH cases, corroborating prior experimental findings regarding its diminished expression patterns. Consequently, *PTGS2* is positioned as a promising biomarker candidate based on our results, yet its diagnostic applicability and mechanistic foundations await definitive verification.

*MCL1* downregulation in IPAH constitutes a paradox: although depleting this antiapoptotic regulator should intuitively foster apoptosis (a process potentially beneficial for mitigating remodeling), its documented role in maintaining smooth muscle cell viability under hypoxia^[32]^ implies a more nuanced, context-dependent function that demands investigation in IPAH-relevant models. While Ibe et al^[32]^ found that *MCL1* knockout significantly increased the apoptosis of human pulmonary artery smooth muscle cells under hypoxic conditions, and that adenosine monophosphate–activated protein kinase α2 (*AMPK α2*) promotes the survival of human pulmonary artery smooth muscle cells by maintaining *MCL1* expression, we observed *MCL1* downregulation. This dichotomy confirms *MCL1*’s context-dependent actions in pulmonary hypertension, warranting further research for definitive mechanistic insight. In addition, some studies have concluded that the ablative expression and/or function of *MCL1* is sufficient to promote apoptosis among neutrophils and thus reduce the inflammatory response.^[33,34]^ Within the context of IPAH pathophysiology, the divergence between these reports and our findings could be reconciled by 2 factors: the overlooked *MCL1L/S* isoform ratio,^[35]^ and inherent inter-individual variability among study subjects. Because there are few studies on the association between *MCL1* and IPAH, it is unclear whether *MCL1* is closely linked to IPAH. Further research is required to address this issue.

The potential involvement of *HUWE1, RNF6, KMT2A, KMT2C*, and *KAT6A* in IPAH remains largely unexplored. Given their known roles in modulating cell proliferation, survival, and epigenetic regulation,^[36–44]^ these factors represent high-priority targets for subsequent mechanistic research into IPAH pathogenesis. Their simultaneous upregulation further implies the existence of a coordinated regulatory network potentially underlying transcriptional and proliferative alterations in this disease.

This study has several limitations that should be adequately addressed in future research. First, due to the difficulty in obtaining human lung tissue samples, this study relied on transcriptomic data from public databases such as GEO. Several inherent data limitations warrant consideration, including finite cohort sizes, technical heterogeneity across platforms, variability in preprocessing, and insufficient clinical metadata (age, sex, medication, severity), collectively preventing robust adjustment for potential confounding factors. Second, bioinformatics-based analytical approaches are exploratory in nature and lack unified standardized procedures; therefore, the findings require further validation through subsequent experiments. However, due to financial constraints, we were unable to perform functional experiments to verify the above discoveries, and this work urgently calls for in-depth exploration by future researchers.

Notably, VEGFA’s network connectivity suggests potential functional crosstalk with other hub genes, a possibility requiring experimental verification. Nevertheless, the specific regulatory mechanisms involved still require further experimental validation. On the other hand, the biological roles of the hub genes identified in this study, including HUWE1, RNF6, KMT2A, KMT2C, and KAT6A, in PAH are currently poorly documented, and their specific mechanisms remain unclear. In conclusion, the results of this study are primarily hypothesis-generating and intended to provide new clues for research into the molecular mechanisms of PAH.

This study establishes a basis for several essential future investigations. First, the mechanistic roles of identified hub genes, including VEGFA, MDM2, and PTGS2, must be functionally assessed using both in vitro and in vivo pulmonary hypertension models. Second, the biomarker potential of these candidates warrants evaluation in prospective, independent cohorts with detailed clinical annotations to account for confounding variables. Finally, integration of multi-omics datasets, such as proteomic profiles from patient serum or epigenomic data from lung tissue, could help clarify upstream regulatory networks and downstream pathways, enabling a more comprehensive systems biology perspective on IPAH pathogenesis.

## 5. Conclusion

While our approach was computational in nature, the 10 hub genes identified present promising candidates for future exploration in IPAH prevention, diagnostic strategies, and therapeutic development. These findings should be interpreted as generating hypotheses that necessitate confirmation through rigorous experimental validation to establish their biological significance. Notably, VEGFA emerged as a particularly intriguing and unanticipated differentially expressed gene, with its downregulation potentially representing a distinctive feature of IPAH among pulmonary hypertension subtypes.

## Author contributions

**Conceptualization:** Yingchuan Zhou, Wenze Deng.

**Data curation:** Yingchuan Zhou.

**Formal analysis:** Yingchuan Zhou.

**Funding acquisition:** Wenze Deng.

**Investigation:** Ailing Ou.

**Methodology:** Ailing Ou.

**Project administration:** Lifang Xu.

**Resources:** Lifang Xu.

**Software:** Ailing Ou.

**Supervision:** Lifang Xu, Wenze Deng.

**Validation:** Shuilian Gan.

**Visualization:** Shuilian Gan.

**Writing – original draft:** Yingchuan Zhou.

**Writing – review & editing:** Wenze Deng.
